# Technological Vanguard: the outstanding performance of the LTY-CNN model for the early prediction of epileptic seizures

**DOI:** 10.1186/s12967-024-04945-x

**Published:** 2024-02-16

**Authors:** Yang Yang, Tianyun Luan, Zhangjun Yu, Min Zhang, Fengtian Li, Xing Chen, Fei Gao, Zhijun Zhang

**Affiliations:** 1 School of Electronic Information Engineering, Changchun University of Science and Technology, Changchun, 130022 China; 2 School of Artificial Intelligence, Changchun Information Technology College, Changchun, 130103 China; 3 Department of Cardiology, JiLin Province FAW General Hospital, Changchun, 130011 Jilin China; 4 School of Artificial Intelligence Industry, Changchun University of Architecture, Changchun, 130607 China; 5 Nuctech Company Limited, Beijing, 100084 China; 6 Jilin Province Advanced Control Technology and Intelligent Automation Equipment R &D Engineering Laboratory, Changchun, 130022 China

**Keywords:** LTY-CNN Model, Multihead attention mechanism, Automated epilepsy detection, Quantized neural network, EEG

## Abstract

**Background:**

Epilepsy is a common neurological disorder that affects approximately 60 million people worldwide. Characterized by unpredictable neural electrical activity abnormalities, it results in seizures with varying intensity levels. Electroencephalography (EEG), as a crucial technology for monitoring and predicting epileptic seizures, plays an essential role in improving the quality of life for people with epilepsy.

**Method:**

This study introduces an innovative deep learning model, a lightweight triscale yielding convolutional neural network” (LTY-CNN), that is specifically designed for EEG signal analysis. The model integrates a parallel convolutional structure with a multihead attention mechanism to capture complex EEG signal features across multiple scales and enhance the efficiency achieved when processing time series data. The lightweight design of the LTY-CNN enables it to maintain high performance in environments with limited computational resources while preserving the interpretability and maintainability of the model.

**Results:**

In tests conducted on the SWEC-ETHZ and CHB-MIT datasets, the LTY-CNN demonstrated outstanding performance. On the SWEC-ETHZ dataset, the LTY-CNN achieved an accuracy of 99.9%, an area under the receiver operating characteristic curve (AUROC) of 0.99, a sensitivity of 99.9%, and a specificity of 98.8%. Furthermore, on the CHB-MIT dataset, it recorded an accuracy of 99%, an AUROC of 0.932, a sensitivity of 99.1%, and a specificity of 93.2%. These results signify the remarkable ability of the LTY-CNN to distinguish between epileptic seizures and nonseizure events. Compared to other existing epilepsy detection classifiers, the LTY-CNN attained higher accuracy and sensitivity.

**Conclusion:**

The high accuracy and sensitivity of the LTY-CNN model demonstrate its significant potential for epilepsy management, particularly in terms of predicting and mitigating epileptic seizures. Its value in personalized treatments and widespread clinical applications reflects the broad prospects of deep learning in the health care sector. This also highlights the crucial role of technological innovation in enhancing the quality of life experienced by patients.

## Introduction

Epilepsy is a global health issue, with approximately 60 million people affected worldwide according to the World Health Organization [1]. Patients with epilepsy experience sudden abnormal neural electrical activity, leading to seizures with varying degrees of severity, ranging from minor distractions to a complete loss of consciousness [2]. The unpredictability of this disease imposes significant physical and psychological burdens on patients [3], limiting their social participation and quality of life. EEG, as an effective tool for monitoring electrical activity in the brain, has become an indispensable part of epilepsy research [4]. With the advent of deep learning and other advanced machine learning technologies, the levels of EEG signal analysis and understanding have significantly improved [5]. By extracting key features from these signals and effectively classifying them, researchers can better predict epileptic seizures, thereby offering more timely interventions for patients [6–8].

In recent years, technological advancements in the field of artificial intelligence have brought new hope to patients. Specifically, modern predictive models, through multiscale feature extraction and multidomain feature analysis, are able to comprehensively capture the complexity of EEG signals [9]. These models not only improve the accuracy of the obtained predictions but also expand our understanding of epilepsy and its seizure mechanisms. The approach proposed by Jee et al. [10] utilizes the synchronous extraction transform and a 1D-CNN, focusing on the extraction of features from time series data. While this method may perform well on specific datasets, its performance stability across different application settings can be challenging to maintain, limiting its broad applicability. Building on the work of Jee et al., the CNN-Bi-LSTM model proposed by Ma et al. [11] was specifically designed to capture and understand the long-term dependencies contained in time series data. This approach, which combines convolutional neural networks and bidirectional long short-term memory networks, has an enhanced ability to recognize complex temporal patterns but also increases the computational burden imposed on the model. Expanding further into multidimensional feature extraction, Lu et al. [12] developed the CBAM -3D CNN-LSTM model, which integrates spatial and temporal features by using a 3D CNN to capture spatial attributes and LSTM to capture temporal attributes. However, this method may encounter limitations when handling multiscale features, particularly in terms of the complexity of EEG data. Guo et al. [13] proposed the CLEP method, employing a spatiotemporal-spectral network for epilepsy prediction; this approach is a highly advanced feature extraction solution. Nonetheless, the complexity of its model structure might impact the interpretability of its results, especially in scenarios requiring high explanatory power. As the depth and complexity of technology further increased, Wang et al. [14] used multibranch dynamic multigraph convolution and channel weighting strategies to handle the multidomain dynamics in EEG signals. While this deep structure is powerful, it may lead to increased technical complexity and maintenance costs [15]. When considering specific data processing needs, Liu et al. [16] employed a power spectral density parametrization method for the classification and prediction of epileptic signals; their approach exhibited significant effectiveness in separating periodic and aperiodic components [17]. However, for EEG signals with high noise levels, this method may face limitations. Shyu et al. [18] achieved notable EEG epilepsy detection results with their parameter-optimized Inception-based end-to-end CNN model, but such end-to-end models might encounter flexibility and adjustability challenges in practical applications [19].

In the realm of epilepsy prediction, although many of the existing models are powerful, they often struggle to balance the parameter scale, search capability, and processing speed, particularly in real-time processing cases and resource-constrained environments [20, 21]. To address these issues, this study introduces a model named the lightweight triscale yielding convolutional neural network (LTY-CNN), which is distinguished by its lightweight architectural design. The LTY-CNN employs a unique parallel convolutional structure to capture EEG signal features across multiple scales, ensuring the comprehensive integration of key information. Through quantization techniques, the model significantly reduces the number of necessary parameters, thereby enhancing its computational efficiency and decreasing its memory requirements. An integrated multihead attention mechanism further boosts the ability of the model to process time series data, improving its prediction accuracy. This innovative design enables the LTY-CNN to maintain exceptional performance even in environments with limited computational resources and offers distinct advantages in terms of model interpretability and maintainability.

The exceptional performance of the LTY-CNN model was validated through test results obtained on different datasets. On the SWEC-ETHZ dataset, the LTY-CNN achieved near-perfect test accuracy and AUROC values, reaching 99.9% and 0.99, respectively, with a test sensitivity of 99.9% and a notably high test specificity of 98.8%. These results demonstrate the extraordinary ability of the LTY-CNN to distinguish between epileptic seizures and nonseizure events with very high accuracy. On the CHB-MIT dataset, the LTY-CNN also displayed efficient performance, with a test accuracy of 99%, an AUROC of 0.932, test sensitivity of 99.1%, and test specificity of 93.2%. These metrics not only prove the robustness of the LTY-CNN in terms of handling datasets with varying characteristics but also establish its status as a reliable epilepsy prediction tool in clinical settings.

## Methods

**Fig. 1:**
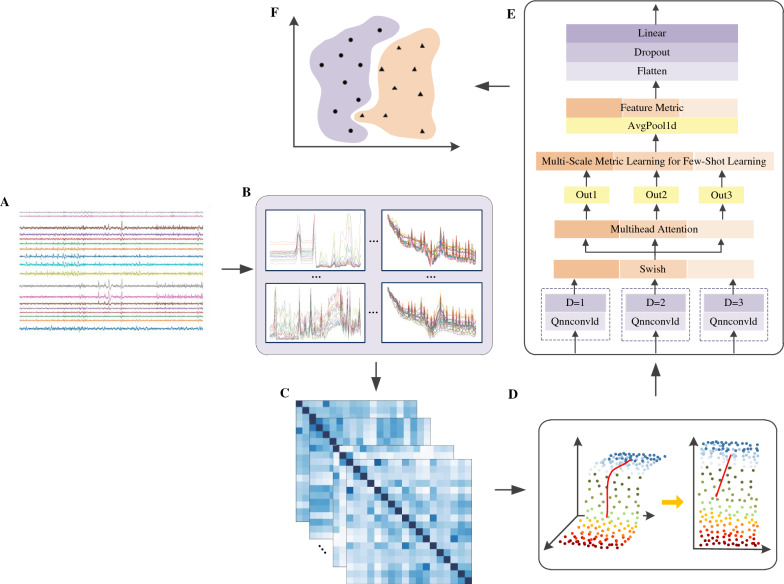
**A** displays a schematic of the EEG signal, detailing how waveforms vary in different states of brain activity. The figure also shows the differences in EEG waves between normal and abnormal states; **B** is a chart of time-domain and spectral analysis, with a focus on signal intensity in alpha and beta waves; **C** presents a channel correlation analysis in different patients’ epilepsy conditions, showing the correlation of EEG signals between different brain region channels during epileptic seizures in various patients. This is represented through color coding and line thickness to indicate the strength of correlation between channels, and how this correlation changes from normal to seizure states; **D** depicts the process of dimension reduction using principal component analysis (PCA), explaining how PCA is utilized for dimension reduction in EEG data. It shows the transformation of data from a high-dimensional space to a low-dimensional space, and the key information preserved in this process; **E** presents our model structure, illustrating the architecture used for analyzing EEG signals, including various processing layers, network architecture, and its outputs; Finally, **F** illustrates the implementation of the classification process, showing how the model classifies EEG signals, such as distinguishing between normal and abnormal signals

This study adheres to the reporting guidelines of the Global Epilepsy Report. The overall workflow of the research is illustrated in Fig. 1.

The extraction strategy is illustrated in Algorithm 1.

**Algorithm 1:**
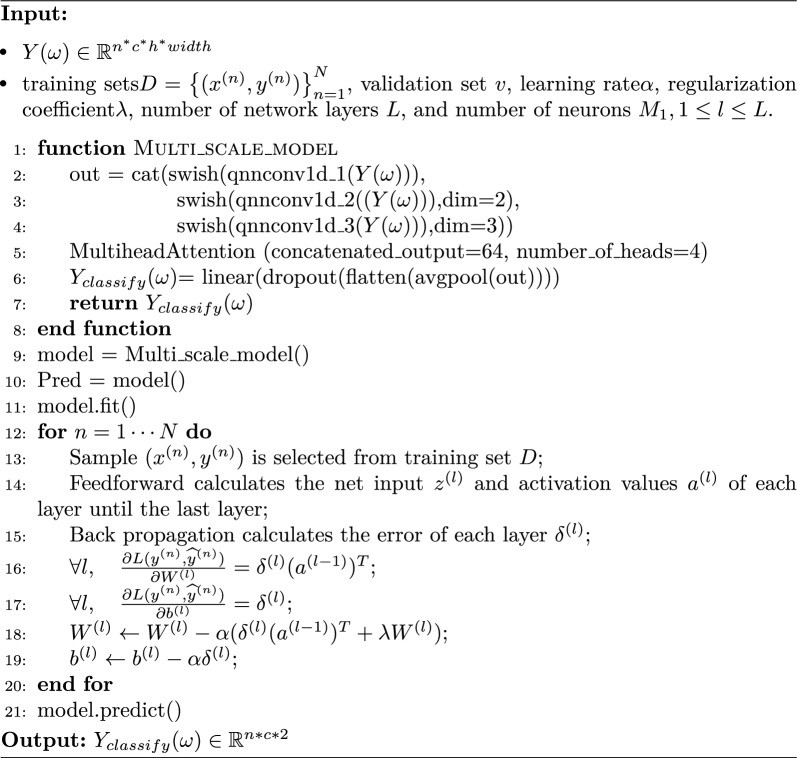
Multiscale epilepsy feature extraction

### Datasets

**Fig. 2:**
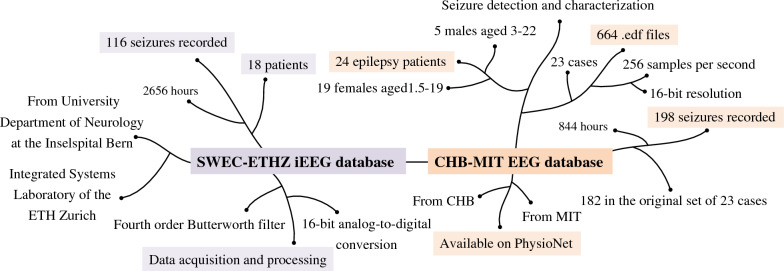
In this figure, we provide a detailed overview of the SWEC-ETHZ and CHB-MIT datasets, including the structure, characteristics, and utilization of each dataset

The SWEC-ETHZ iEEG Database was created through a collaboration between the Sleep-Wake-Epilepsy-Center (SWEC) at the University Hospital of Bern and ETH Zurich. It provides 2656 h of continuous intracranial electroencephalography data from 18 patients with drug-resistant epilepsy, including 116 epileptic seizure events. The data has been anonymized to protect patient privacy. The iEEG signals have been band-pass filtered and sampled at either 512 Hz or 1024 Hz. All recordings have been visually inspected by experts to identify the start and end points of epileptic seizures and to exclude channels affected by interference. This dataset fills a gap in the availability of public long-term continuous iEEG epilepsy datasets and is used for assessing the performance of epilepsy detection algorithms. Second, the CHB-MIT EEG database was created via a collaboration between Boston Children’s Hospital and the Massachusetts Institute of Technology. It offers long-term monitored EEG recordings of epileptic seizures in 24 children aged between 1.5 and 22 years (including 5 males and 19 females). These recordings include detailed annotations of 182 epileptic seizures, covering 23 cases, each of which consists of 9 to 42 continuous.edf files. All signals were sampled at a rate of 256 samples per second, with a 16-bit resolution. These data were used to further validate the effectiveness of the model for predicting epileptic seizures. The data collection processes employed for both databases adhered to the principles of the Helsinki Declaration and received approval from the respective institutions’ ethics committees, ensuring the ethical compliance of this research. Information about the datasets is depicted in Fig. 2.

### Data preprocessing

In the data preprocessing phase of our study, we carefully selected data from all participants in the SWEC-ETHZ iEEG database, except for case 9. Upon conducting a careful evaluation, we found that the data from case 9 did not align with our established processing methods and had quality issues; hence, we decided to exclude it from the subsequent analysis. For the other participants, their data included electrical brain activity recordings varying from 8 to 18 h, accumulating over 202 h of valuable electroencephalographic information. These data provided a rich foundation for our study, offering us the opportunity to delve deeper into exploring predictive models for epileptic seizures. When processing the CHB-MIT EEG database, we adopted similar selection criteria. Specifically, we excluded data from cases 12, 20, and 24 due to their incompatibility with our processing methods and their failure to meet our high data quality standards. For the remaining patients in the database, we focused on analysing the data derived from the first 18 internationally recognized standard epilepsy assessment channels for each patient, ensuring the global applicability and standardization of our research findings. In a unified data processing workflow, all signals were meticulously downsampled, ultimately reducing the sampling rate of the original signals to 256 Hz. This step aimed to balance the data resolution and computational efficiency while maintaining sufficient information for supporting the accuracy of the subsequent analysis. Concurrently, we set a 64-s window size as the basic unit for batch processing, allowing us to maintain the temporal integrity of the data while providing an appropriate amount of input data for the machine learning models. Through these meticulous data preprocessing steps, we established a solid foundation for developing and validating algorithms to predict epileptic seizures, ensuring the accuracy and reliability of our research.

### Feature extraction

**Fig. 3:**
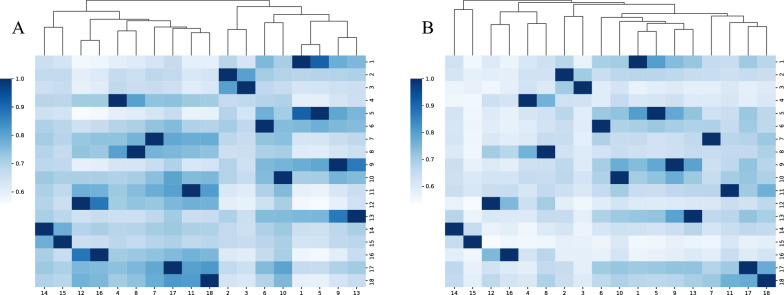
The correlation analysis and clustering results obtained for the 18 tested channels

In this study, we conducted a comparative analysis of EEG data from male and female participants, which are presented in Fig. 3A (female) and Fig. 3B (male), respectively. Figure 3A reveals the correlations between 18 EEG channels in female participants, with some notable correlations, such as a high value of 0.914 between channels 1 and 5, indicating a high degree of similarity in the brain activities recorded by these channels, possibly reflecting the cooperative action of adjacent brain areas. In Fig. 3B, based on the data, we observed a different pattern of correlations between EEG channels in male participants. For example, the correlation between channels 1 and 12 in males is 0.572, slightly lower than in females, which may reveal characteristics of brain network connections under gender differences. On the other hand, the extremely high correlation (0.881) between channels 16 and 12 is more pronounced in the male sample, suggesting that certain brain regions may exhibit more significant cooperative activities in males.

These findings reveal shared features and differences between males and females in specific EEG channel groups, which may reflect gender-specific patterns of neural network connections. Through this detailed comparative analysis, we can gain a deeper understanding of how gender affects the function and structure of the brain. This discovery provides a new perspective for exploring the role of gender in the field of neuroscience.

During the preprocessing phase, we implemented precise filtering measures. A band-stop filter was used to eliminate the frequency components between 117 to 123 Hertz and 57 to 63 Hertz, targeting noise potentially introduced by power lines and other electrical devices. The band-stop filter is represented as:

$$\begin{aligned} H_{bp}(f) = H(f) \cdot (1 - H_{bs}(f)) \end{aligned}$$

Here, $H(f)$ is the transfer function of the filter, and $H_{bs}(f)$ is the transfer function of the stop band in the band-stop filter, which was designed to attenuate the signal to a very low level within a specific frequency range. A high-pass filter was applied to exclude all frequencies below 1 Hertz, reducing signal interference caused by slow-wave activity. The high-pass filter is represented as:

$$\begin{aligned} H_{hp}(f) = \frac{f}{f + f_c} \end{aligned}$$

where $f$ is the frequency of the signal, and $f_c$ is the cutoff frequency of the high-pass filter, which was set to 1 Hertz in this work.

The signal processing work involved segmenting continuous EEG recordings into 64-s windows, resulting in each data segment containing 16,384 sampling points. Utilizing these data fragments, we generated spectrograms with precise temporal resolutions, further enhancing our ability to recognize the dynamics of brain electrical activity. Each spectrogram included 127 temporal resolution units and 114 frequency resolution units, allowing us to meticulously analyse time series data and capture subtle frequency distribution variations.

In the in-depth analysis of the data and the model construction process, we further employed PCA to achieve effective dimensionality reduction for the data. The fundamental principle of PCA is to transform the original data into a new coordinate system through an orthogonal transformation, where the first dimension of this new system has the maximum data variance. The specific PCA transformation process is represented as follows:

$$\begin{aligned} {\textbf {Y}} = {\textbf {XW}} \end{aligned}$$

Here, ${\textbf {X}}$ is the mean-centred original data matrix, ${\textbf {W}}$ is a matrix composed of the principal components extracted from the original data, and ${\textbf {Y}}$ denotes the transformed data, which include the main features and information. By applying singular value decomposition techniques, the original data were projected into a low-dimensional space composed of 64 principal axes. This process captured the core variables of the signals, ensuring precise information extraction and an optimized balance in the dimensionality reduction technique.

$$\begin{aligned} {\textbf {X}} = {\textbf {UDV}}^T \end{aligned}$$

${\textbf {U}}$ and ${\textbf {V}}$ are orthogonal matrices, while ${\textbf {D}}$ is a diagonal matrix, the diagonal elements of which are singular values, representing the variance of the data in the direction of each principal component. Through a systematic cross-validation, we confirmed that these selected principal components sufficiently captured the primary variability of the signals, thereby verifying that a delicate balance between information loss and data simplification had been achieved. We adopted an innovative heuristic overlapping sampling technique.

### Parallelized architectural framework for EEG analysis

The distinct feature of this architecture is its parallel use of wide, medium, and narrow convolution kernels to process different features within the same EEG signal input, thereby capturing varying signal characteristics. These three convolution kernels with different sizes worked in parallel, complementing each other to form a comprehensive feature extraction strategy. The outputs of the parallel convolution layers were integrated to form a composite feature representation:

$$\begin{aligned} {\textbf {F}}_{\text {total}} = \text {Concat}[\text {Act}({\textbf {F}}_{\text {wide}}), \text {Act}({\textbf {F}}_{\text {medium}}), \text {Act}({\textbf {F}}_{\text {narrow}})] \end{aligned}$$

Here, $\text {Act}$ is the activation function, which was used to introduce nonlinearity and assist in capturing more complex features.

The parallel architecture optimized the computational process of the convolution layers, reducing the time complexity of the model. In traditional serial convolution networks, the time complexity of feature extraction $O_{\text {serial}}$ is the sum of the time complexities of different layers:

$$\begin{aligned} O_{\text {serial}} = \sum _{i=1}^{N} O_{i} \end{aligned}$$

However, in the parallelized design, the simultaneous execution of multiple convolution operations significantly reduces the temporal complexity $O_{\text {parallel}}$:

$$\begin{aligned} O_{\text {parallel}} = O_{\text {max}} \end{aligned}$$

Here, $O_{\text {max}}$ is the maximum time complexity of any single convolution operation. With the support of multicore hardware, the theoretically achievable processing time approaches the following:

$$\begin{aligned} T_{\text {efficient}} \approx O_{\text {max}} \end{aligned}$$

**Fig. 4:**
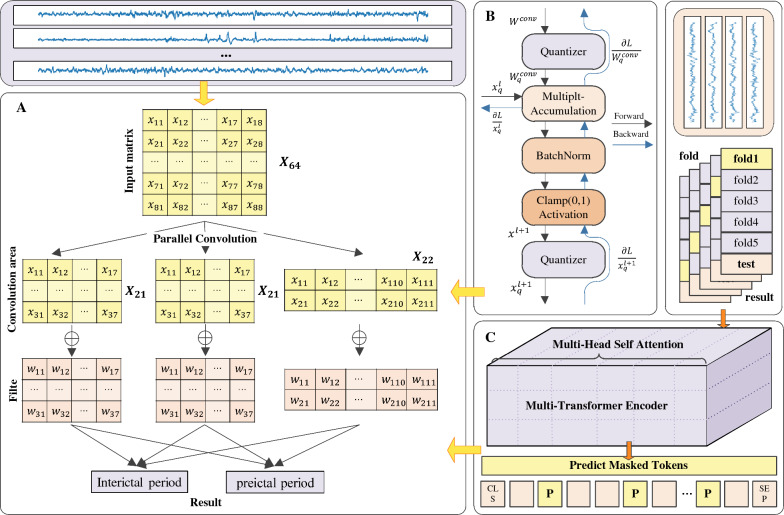
**A** Shows the complete feature extraction process utilized in this paper. During this process, **B** illustrates how the burden imposed on the model is effectively alleviated through the quantization network strategy. In **C**, we employ the multihead attention mechanism to precisely extract and analyse data information

This specific strategy is depicted in Fig. 4A. Through such a parallel convolution design, we could significantly enhance the processing speed while maintaining the comprehensive feature extraction ability of the model for EEG signals, making the model more suitable for real-time analysis scenarios.

### Enhanced feature extraction with quantized convolution and dilation

Quantized convolution is a technique for optimizing neural networks to fit resource-constrained environments. It is achieved by reducing the precision levels of weights $W$ and activations $A$ from 32-bit floating-point numbers to $n$-bit fixed-point numbers. This process can be described by the quantization function $Q$, which maps continuous input values to discrete quantization levels. The quantization function can be defined as:

$$\begin{aligned} Q(x) = \Delta \cdot \left\lfloor \frac{x}{\Delta } + \frac{1}{2} \right\rfloor \end{aligned}$$

Here, $\Delta$ is the quantization step, which determines the granularity of quantization, is typically related to the quantization bit width $n$ and can be determined by the following formula:

$$\begin{aligned} \Delta = \frac{2 \cdot \max (|x|)}{2^n} \end{aligned}$$

$\max (|x|)$ represents the maximum absolute value of the input $x$, ensuring that the quantized values can cover the dynamic range of the input values. The error $E$ in the quantization process can be quantified by the expected value of the quantization error:

$$\begin{aligned} E = {{\mathbb {E}}}[|x - Q(x)|] \end{aligned}$$

To reduce the error introduced by quantization, quantization-aware training is often employed. This simulates the effects of quantization during the training process, adjusting the network parameters to adapt to the quantized representation. This can be achieved by introducing the gradient of the quantization error, thereby considering the impact of quantization during backpropagation:

$$\begin{aligned} \frac{\partial E}{\partial x} = \frac{\partial }{\partial x} {{\mathbb {E}}}[|x - Q(x)|] \end{aligned}$$

Furthermore, the output $O$ of the quantized convolutional layer can be represented as the convolution of the quantized weights $W_q$ and activations $A_q$:

$$\begin{aligned} O = Q(A) * Q(W) \end{aligned}$$

Here, $*$ denotes the convolution operation. In this way, quantized convolutional layers can significantly reduce the storage requirements and computational complexity of the model while maintaining the performance of the network.

In our quantized convolutions, we introduce dilated convolution, a special type of convolutional operation that expands the receptive field by introducing spaces between adjacent elements in the convolutional kernel, without increasing the imposed computational burden. This strategy allows the network to capture a broader range of contextual information at deeper levels without significantly increasing the number of required parameters. Dilated convolution introduces a dilation rate $d$, which defines the spacing between the elements in the convolutional kernel. Thus, the output $O_d$ of a dilated convolution can be calculated using the following formula:

$$\begin{aligned} O_d(i, j) = \sum _m \sum _n A(i + d \cdot m, j + d \cdot n) \cdot W(m, n) \end{aligned}$$

Here, $d$ is the dilation rate, and $m$ and $n$ are the indices of the convolutional kernel $W$. When $d = 1$, the dilated convolution degenerates to a standard convolution. As $d$ increases, the receptive field $R$ also expands, and this is calculated as:

$$\begin{aligned} R = k + (k - 1) \cdot (d - 1) \end{aligned}$$

where $k$ is the size of the convolutional kernel. Dilated convolution increases the receptive field in this manner rather than by increasing the size of the convolutional kernel or the depth of the network, thereby enhancing the network’s understanding of the input data without significantly increasing its computational complexity.

Combined with quantized convolution, dilated convolution can achieve more effective feature extraction effects in quantized networks. The output $O_q$ of the quantized convolutional layer can be modified through dilated convolution as follows:

$$\begin{aligned} O_q(i, j) = \sum _m \sum _n Q(A(i + d \cdot m, j + d \cdot n)) \cdot Q(W(m, n)) \end{aligned}$$

This specific strategy is depicted in Fig. 4B. Through the combination of dilated and quantized convolutions, the ability of the network to process large-scale input data can be enhanced while maintaining accuracy.

### Capturing time series with multihead attention

In this section, we explore the application of a multihead attention mechanism for improving epilepsy prediction models. The introduction of the multihead attention mechanism aims to enhance the ability of the constructed model to capture time series data. The multihead attention layer in the model is configured with four attention heads, each of which is capable of independently focusing on different features of the input data. The embedding dimensionality of each head is set to 64, which is a dimensional choice that helps capture sufficient feature information while maintaining model complexity.

The operation process of the multihead attention layer can be represented as:

$$\begin{aligned} \text {MultiHead}(Q, K, V) = \text {Concat}(\text {head}_1,\ldots , \text {head}_N)W^O \end{aligned}$$

where the computation of each head is:

$$\begin{aligned} \text {head}_i = \text {Attention}(QW_i^Q, KW_i^K, VW_i^V) \end{aligned}$$

The attention function is defined as:

$$\begin{aligned} \text {Attention}(Q, K, V) = \text {softmax}\left( \frac{QK^T}{\sqrt{d_k}}\right) V \end{aligned}$$

Here, $Q$, $K$, and $V$ represent the query, key, and value matrices, respectively, and $W_i^Q$, $W_i^K$, $W_i^V$, and $W^O$ are learnable parameter matrices.

The specific strategy is depicted in Fig. 4C. Through this design, the model is expected to capture the temporal dynamics within EEG signals in a finer manner, especially at critical moments when predicting epileptic seizures. The parallel processing ability of the multihead attention mechanism enables the model to potentially capture richer temporal information from different representation spaces, which is crucial for understanding the complexity of EEG signals. The inclusion of the multihead attention mechanism provides prediction accuracy advantages, particularly in terms of capturing subtle signal changes that may indicate impending epileptic seizures.

## Results and discussion

### Experimental environment

The hardware and software environment utilized in this experiment is shown in Table 1.

**Table 1:** Experimental hardware and software environment

Environments	Attributes
Processor	Intel(R) Core(TM) i9-10920X CPU@3.50GHz
Disc	1T SSD
RAM	256 GB
Operation system	Windows 11
Development Languages	Python 3.9
Deep learning frameworks	Pytorch 1.10
GPU	NVIDIA GeForce RTX3090

### Evaluation criteria

In this study, to comprehensively evaluate the performance of the LTY-CNN model, we employed a series of evaluation metrics. These metrics included the accuracy (Acc), AUROC (AUC), sensitivity (Sen), and specificity (Spe) of the model.

The mathematical formulas corresponding to each metric are presented below:

$$\begin{aligned}{} & {} Acc = \frac{TP + TN}{TP + TN + FP + FN} \\{} & {} Sen = \frac{TP}{TP + FN}\\{} & {} Spe = \frac{TN}{TN + FP} \\{} & {} TPR = \frac{TP}{TP + FN} \\{} & {} FPR = \frac{FP}{FP + TN} \end{aligned}$$

In these metrics, TP, TN, FP, and FN represent the numbers of true-positive, true-negative, false-positive, and false-negative predictions made by the model, respectively. The AUC range is from 0 to 1. The closer the AUC is to 1, the better the classification performance of the model. The calculation of AUC is typically achieved through numerical integration methods.

### Prediction performance evaluation

Here, we delve into an application of the deep learning-based LTY-CNN model involving the prediction of epileptic seizures, specifically targeting two different datasets: SWEC-ETHZ and CHB-MIT. Utilizing the fivefold cross-validation method, this model not only reduced the randomness of the results but also significantly lowers the risk of overfitting, thereby greatly enhancing the generalization ability of the model. The test results obtained on the SWEC-ETHZ dataset, as shown in Table 2, reveal the remarkable accuracy of the LTY-CNN model, with an average accuracy rate reaching 99.9%. This near-perfect outcome implies that the model could identify epileptic seizures with extreme precision. Particularly, for the vast majority of patients, both the sensitivity and specificity of the model’s predictions reached 100%, indicating that in these cases, the model could flawlessly distinguish between seizure and nonseizure states.

**Table 2:** Epilepsy detection performance achieved using multiscale feature extraction methods on the SWEC-ETHZ dataset

Patient id	Acc (%)	Sen (%)	Spe (%)	AUC
Pt 01	100.0	100.0	100.0	1.000
Pt 02	100.0	100.0	100.0	1.000
Pt 03	100.0	100.0	100.0	1.000
Pt 04	100.0	100.0	100.0	1.000
Pt 05	100.0	100.0	100.0	1.000
Pt 06	100.0	100.0	100.0	1.000
Pt 07	100.0	100.0	100.0	1.000
Pt 08	100.0	100.0	100.0	1.000
Pt 10	99.5	99.7	80.0	0.899
Pt 11	100.0	100.0	100.0	1.000
Pt 12	100.0	100.0	100.0	1.000
Pt 13	100.0	100.0	100.0	1.000
Pt 14	100.0	100.0	100.0	1.000
Pt 15	99.2	99.2	100.0	0.925
Pt 16	100.0	100.0	100.0	1.000
Pt 17	100.0	100.0	100.0	1.000
Pt 18	100.0	100.0	100.0	1.000
Average	99.9	99.9	98.8	0.990

As shown in Table 3, a subsequent validation conducted on the CHB-MIT dataset revealed that despite a decrease in the specificity achieved for individual patients (e.g., the specificity for Pt 05 and Pt 21 was 80.0%), the overall performance remained robust. The average accuracy was 99.0%, the average sensitivity was 99.1%, and the average specificity slightly decreased to 93.2%. Although the specificity was somewhat lower than before, this did not significantly diminish the overall superior performance of the proposed model. The reduced specificity might point to individual variability in the data, suggesting a need for further personalizing the model parameters or adjusting the model structure to accommodate the characteristics of different patients.

**Table 3:** Epilepsy detection performance achieved using multiscale feature extraction methods on the CHB-MIT dataset

Patient id	Acc (%)	Sen (%)	Spe (%)	AUC
Pt 01	98.6	98.9	91.8	0.929
Pt 02	99.5	99.6	97.2	0.943
Pt 03	99.3	99.3	98.2	0.945
Pt 04	99.9	99.9	100.0	0.950
Pt 05	99.1	99.2	80.0	0.900
Pt 06	98.4	99.0	90.0	0.937
Pt 07	99.5	99.5	86.7	0.906
Pt 08	97.8	98.0	92.7	0.924
Pt 09	99.7	99.8	94.6	0.953
Pt 10	98.1	98.4	90.5	0.923
Pt 11	99.7	99.7	100.0	0.900
Pt 13	99.0	99.1	96.7	0.943
Pt 14	97.5	97.5	99.5	0.914
Pt 15	99.9	99.9	100.0	0.950
Pt 16	97.9	98.3	95.6	0.958
Pt 17	98.7	98.7	100.0	0.918
Pt 18	99.1	99.4	89.1	0.932
Pt 19	99.6	99.7	95.6	0.963
Pt 21	98.8	98.9	80.0	0.887
Pt 22	99.0	99.3	83.3	0.907
Pt 23	99.1	99.5	96.6	0.980
Average	99.0	99.1	93.2	0.932

When analysing the relationships between patients, we observed that even within the same dataset, variations were observed among the accuracy and area under the receiver operating characteristic curve metrics of different patients. This may reflect the diversity of the epileptic seizure biomarkers across individuals. For example, in the CHB-MIT dataset, Pt 04 and Pt 15 demonstrated that the model could achieve near-perfect predictive accuracy in individual cases, while Pt 05 and Pt 21 indicated the need for further adjusting the model when processing data from certain individuals. This variability underscores the importance of personalized medicine, highlighting that even highly efficient models must account for the biological and clinical heterogeneity among patients.

On the SWEC-ETHZ dataset, the model demonstrated astonishing accuracy, achieving an average accuracy of 99.9%. This result is nearly perfect, indicating that the model can identify epileptic seizures with extreme precision. Notably, for the majority of patients, both sensitivity and specificity of the model’s predictions reached 100%, suggesting flawless differentiation between seizure and non-seizure states in these cases. In validation on the CHB-MIT dataset, despite a decrease in specificity for some individual patients, the overall performance remained strong with an average accuracy of 99.0%, an average sensitivity of 99.1%, and a slightly lower average specificity of 93.2%. These outcomes reveal the model’s consistency and adaptability across different datasets, even when faced with data of varying origins and qualities. This capability is crucial for clinical applications, as it implies the model’s adaptability to diverse patient populations and various collection conditions.

Future work will focus on improving the model’s performance for patients with lower specificity in datasets similar to CHB-MIT. We plan to further analyze the unique characteristics in these patients’ data, such as seizure frequency, the complexity of background electrical activity, and their relationship with other clinical parameters. Additionally, we will explore more advanced data preprocessing and augmentation techniques, as well as the use of strategies like transfer learning and meta-learning to enhance the model’s adaptability and robustness.

In the analysis of Table 4, the model demonstrates remarkable consistency and efficacy across genders. The performance in terms of accuracy, sensitivity, and specificity shows negligible differences between females and males, achieving accuracy rates of 98.95% and 98.98%, sensitivity rates of 99.15% and 99.08%, and specificity rates of 93.30% and 93.14%, respectively. Such minimal discrepancies are virtually insignificant, indicating the model’s exceptional generalization capability in epilepsy detection across different genders. The AUC values further reflect nearly identical performance between the genders, reinforcing the LTY-CNN’s effectiveness and precision in handling gender-based analysis.

**Table 4:** Analysis on gender differences in the CHB-MIT experiment

Gender	Acc (%)	Sen (%)	Spe (%)	AUC
Female	98.95	99.15	93.30	0.933
Male	98.98	99.08	93.14	0.929

The data in Table 5 reveal the model’s significant stability and efficiency across various age groups. Whether it be children, teenagers, or adults, the model shows high accuracy and sensitivity across all age brackets, with accuracy rates of 98.78%, 99.10%, and 99.43%, and sensitivity rates of 98.95%, 99.28%, and 99.50%, respectively. Notably, in the adult group, the model attains the highest levels of accuracy and sensitivity. Despite a slightly lower specificity in adults compared to other age groups, the AUC values for all age segments collectively indicate that the model possesses outstanding overall performance across all age categories, proving its effectiveness and consistency in epilepsy detection among patients of varying ages.

**Table 5:** Performance results of different age groups in the CHB-MIT dataset

Age group	Acc (%)	Sen (%)	Spe (%)	AUC
Children (0–12 years)	98.78	98.95	93.03	0.928
Teenagers ( 13–18 years)	99.10	99.28	94.37	0.937
Adults (19 years and over)	99.43	99.50	91.87	0.933

The K-fold cross-validation results obtained for the LTY-CNN model, as shown in Table 6, indicated an exceptionally high overall accuracy, averaging 98.68% with minimal variability (a standard deviation of 0.33%). This demonstrated the high consistency of the model in terms of predicting epileptic seizures. The average sensitivity was 93.24% with a standard deviation of 5.13%, indicating that the detection rate of the model for actual seizures was relatively stable across different validation folds, despite some variability. The average specificity was 98.96% with a standard deviation of 0.74%, showing the high accuracy of the model in terms of excluding nonseizure states. The average AUROC was 0.884 with a standard deviation of 0.10, reflecting the model’s good ability to differentiate between seizure and nonseizure states. The analysis results emphasize the importance of accuracy metrics in the model training and validation process, with different K-fold validation outcomes proving the robust and stable performance of the model with respect to processing EEG signals acquired from various patients.

**Table 6:** K-fold cross-validation model performance for patient 4 in the CHB-MIT dataset

K folds	Acc (%)	Sen (%)	Spe (%)	AUC
K=3	98.3	86.2	98.2	0.976
K=6	98.6	96.1	99.3	0.800
K=9	99.2	94.2	99.0	0.875
K=12	98.6	90.3	98.3	1.000
K=15	98.7	99.4	100.0	0.769

**Fig. 5:**
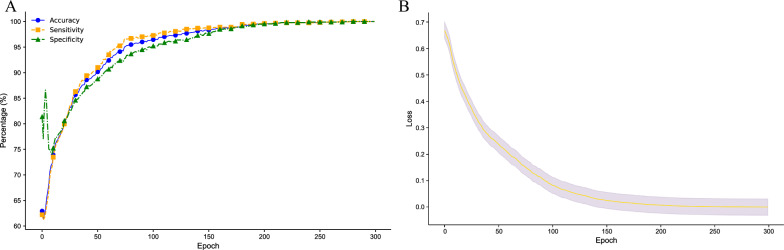
The left graph shows the growth trend curves for the accuracy, sensitivity, and specificity metrics, while the right graph depicts the decreasing trend of the loss value

Taking the growth curve in Fig. 5A as an example, from the initial training cycle to the final cycle, the test performance metrics of the model exhibited significant growth. Specifically, the test accuracy increased from an initial approximate value of 62.94% to 100%, with a growth rate of approximately 58.89%. The test sensitivity improved from approximately 62.21% to 100%, with a growth rate of 60.75%. The test specificity rose from a high initial value of 81.37% to 100%, with a growth rate of 22.89%, which was a smaller change. This may indicate that the model already had a good ability to recognize nonseizure states at the start of the training process. The test AUROC value increased from 0.533 to 1.0, with a high growth rate of 87.49%, showing a significant enhancement in the ability of the model to differentiate between seizure and nonseizure states during the training process. Figure 5B shows the fluctuating loss curve declines observed for different patients. From the declining fluctuation trend, we can see that the model has good convergence. The curve shows a steady downwards trend without significant fluctuations or oscillations, indicating that the model gradually approached the optimal solution during the training process. The 20% error margin of the standard deviation of the loss function is a result derived from experiments conducted by different researchers. This value was determined through data analysis after the model was repeatedly trained and tested by various research teams. We found that using 20% of the standard deviation as a threshold offers an accurate and practical measure, effectively capturing the variability in the model’s performance across different research groups. This approach ensures that our error margin is statistically significant and meaningfully reflects the consistency and stability of the model as evidenced by multiple independent experiments.

**Fig. 6:**
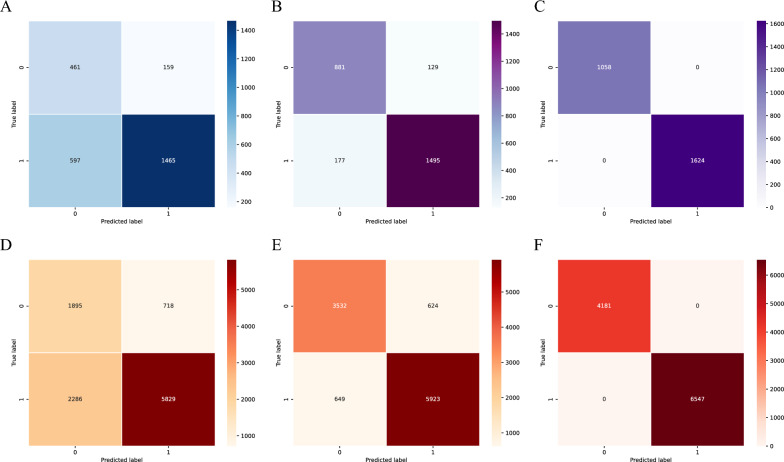
**A**–**C** Show the confusion matrix classification process executed by the model on the test set in detail. **D**–**F** On the other hand, depict a similar confusion matrix classification process undertaken by the model on the training set, demonstrating the performance and classification effectiveness observed during the learning and adaptation process of the model

Our model exhibited outstanding performance on both the test and training sets. Figure 6 presents the changing process of the training confusion matrix produced for our model. Graphs C and F represent the final classification results obtained on the test and training sets, respectively. Our model achieved perfect accuracy and recall rates on both sets, indicating its excellent ability to identify positive and negative samples and demonstrating its superior performance in classification tasks. These results suggest that our model performs well in epilepsy detection tasks, providing reliable support for future clinical applications.

### Comparison among different classifiers

In this study, we compared the performance of the LTY-CNN model with that of several other classifiers in the epileptic seizure detection task. To ensure fairness during the comparison, we referred to various methods from recent studies, including approaches based on PCA and LDA [8], methods utilizing fractal intercept and relative fluctuation indices [9], kernel collaborative representation [10], LDA [11], a seven-layer CNN [12], DLWH [12], MB-dMGC-CWTFFNet [13], the wavelet packet transform- and weighted extreme learning machine-based method [14], CBAM-3DCNN-BiLSTM [15] and SOC-CNN [16]. The specific parameters used for these comparisons are shown in Table 7.

In terms of accuracy, our LTY-CNN model surpassed all other methods with an accuracy rate of 99.5%. This was 4.81% higher than that of the PCA+LDA method reported in the literature [8], 4.6% higher than that of the fractal intercept method in [9], and 2.67% higher than that of the kernel collaborative representation method in [10]. Compared to the LDA method in [11], the LTY-CNN model even exceeded its accuracy by 7.7%.

In terms of sensitivity, the LTY-CNN model tied for first place with the LDA method in [11] and the MB-dMGC-CWTFFNet approach in [13], achieving 100% sensitivity. This means that these methods could detect all true cases of epileptic seizures. However, it should be noted that MB-dMGC-CWTFFNet did not provide specificity data.

In terms of specificity, the LTY-CNN model performed excellently with a specificity of 96%; however, this value was slightly lower than that of the kernel collaborative representation method in [10] and that of the seven-layer CNN in [12], which had specificities of 96.81% and 98.5%, respectively. Compared to the SOC-CNN method in [16], although SOC-CNN achieved 100% specificity, its sensitivity was only 82.35%, which was significantly lower than that of the LTY-CNN model. The LTY-CNN model demonstrated superior performance in terms of accuracy and sensitivity, as well as competitive specificity results. These comparative results highlight the potential and superiority of the LTY-CNN model in the field of epilepsy seizure detection.

**Table 7:** Comprehensive comparative model parameter analysis

Model	Acc (%)	Sen (%)	Spe (%)
PCA+LDA [22]	94.7	94.8	89.1
Fractal intercept and relative fluctuation indices [23]	94.9	91.7	94.9
Kernel collaborative representation [24]	96.8	97.5	96.8
LDA [25]	91.8	100.0	83.6
Seven-layer CNN [26]	97	98.5	98.5
DLWH [26]	95.1	94.3	95.4
MB-dMGC-CWTFFNet [27]	98.4	100.0	-
Wavelet packet transform and weighted extreme learning machine [28]	96.9	95.8	92.2
CBAM-3DCNN-BiLSTM [29]	98.0	98.4	–
SOC-CNN [30]	96.8	82.4	100.0
LTY-CNN	99.5	99.5	96.0

The symbol ‘–’ represents undisclosed model performance metrics data for which no related information has been released at present

### Model efficiency and parameter complexity

In this study, we conducted a detailed comparison between the performance of the LTY-CNN model and that of the existing models in terms of epilepsy prediction with specific parameters, as shown in Table 8. The LTY-CNN model demonstrated exceptional performance, especially in terms of accuracy; its value of 99.5% was only slightly lower than that of the best-performing 2D-CNN model (by 0.5%). Compared to the TSKCNN and another 2D-CNN model, LTY-CNN surpassed them in accuracy by 1.5%, highlighting the LTY-CNN’s significant advantage in prediction precision.

In terms of sensitivity, a key metric, the performance of the LTY-CNN was particularly notable at 99.5%, which was significantly higher than those of SOD-CNN and LRCN, exceeding them by 10.4% and 15.5%, respectively. This achievement not only reflects the strong ability of the LTY-CNN to accurately identify epileptic seizures but also highlights its potential value in clinical applications. Although the specificity performance of the LTY-CNN was slightly lower than that of SOD-CNN and LRCN, given its excellent performance in other key metrics, this difference does not diminish its overall outstanding performance.

The LTY-CNN, while maintaining high performance, significantly reduced the complexity of the model. It has only 24,506 parameters, which is approximately 99.91% less than that required the TSKCNN with the highest number of parameters and approximately 76.8% and 50.6% less than those of the SOD-CNN and the smallest 2D-CNN, respectively. This significant advantage suggests that the LTY-CNN has a considerable advantage in terms of computational efficiency and deployment, making it especially suitable for resource-constrained clinical settings.

**Table 8:** Comparative analysis of the magnitudes and performance indices of the tested models

Method	Parameters	Acc (%)	Sen (%)	Spe (%)
2D-CNN [30]	49,560	98.2	82.7	88.2
TSKCNN [31]	28,459,615	98.0	96.0	99.0
LRCN [32]	9,695,012	99.0	84.0	99.0
SOD-CNN [33]	105,538	99.6	89.1	99.7
2D-CNN [34]	10,304,467	100.0	–	–
2D-CNN [34]	106,388	98.0	–	–
LTY-CNN	24,506	99.5	99.5	96.0

The symbol ‘–’ represents undisclosed model performance metrics data for which no related information has been released at present

**Fig. 7:**
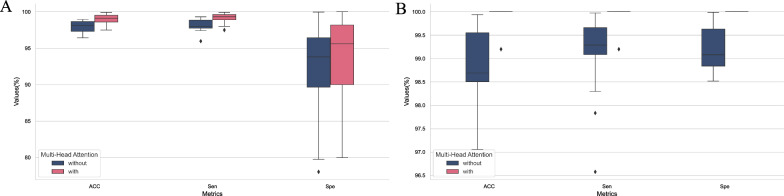
The left graph visually compares the impacts of adding the multihead attention mechanism on the accuracy, sensitivity, and specificity attained for all patients in the CHB-MIT dataset, clearly presenting performance improvements. The right graph conducts a similar analysis on the SWEC-ETHZ dataset, displaying the comparative effects of the multihead attention mechanism across different datasets

In our model, although the introduction of the multihead attention mechanism led to an increase in the number of model parameters, which seems to contradict our original intention of pursuing a lightweight design, it indeed improved the overall performance of the model to a certain extent (by approximately 1% to 2%). The performance improvement achieved on the utilized dataset is illustrated in Fig. 7. Given this performance enhancement, these additional parameters are acceptable to us.

## Conclusion

This study successfully developed a novel deep learning model, the LTY-CNN, which was specifically designed to enhance the accuracy of epilepsy detection. The core innovation of the LTY-CNN lies in its lightweight architecture and multiscale feature extraction ability; it integrates parallel convolutional structures with a multihead attention mechanism, effectively capturing the complex and dynamic changes exhibited by EEG signals. This design not only improves the operational efficiency of the model but also maintains its good interpretability and maintainability, making it an ideal choice in environments with limited computational resources.

In experimental tests, the LTY-CNN demonstrated high accuracy and AUROC metrics on the SWEC-ETHZ and CHB-MIT datasets, signifying its significant advantage in distinguishing between epileptic seizures and nonseizure events. Compared to the existing epilepsy detection methods, the LTY-CNN not only yielded accuracy improvements but also performed exceptionally in terms of sensitivity and specificity, which are particularly crucial for enhancing the effectiveness of epilepsy management.

The outcomes of this study hold significant clinical value for the diagnosis and treatment of epilepsy, and they offer a new perspective for handling complex biological signals using deep learning technology. The successful implementation and validation of the LTY-CNN model suggest that deep learning techniques will play an increasingly vital role in the medical and health care fields, particularly in EEG analysis and neurological disorder diagnosis. In the future, through further optimization and customization, this type of model will play a significant role in personalized medicine and broader clinical applications, bringing more precise and efficient diagnostic and treatment options to patients with epilepsy.

## Data Availability

The SWEC-ETHZ and CHB-MIT datasets mentioned in this paper are both public datasets. They can be downloaded from the following URLs: (1) SWEC-ETHZ dataset: http://ieeg-swez.ethz.ch/#:~:text=%23%20%E3%80%900%E2%80%A0The%20SWEC,swez.ethz.ch%E3%80%91. (2) CHB-MIT dataset: https://physionet.org/content/chbmit/1.0.0/
